# Alterations in Neural Networks During Working Memory Encoding Related to Cognitive Impairment in Temporal Lobe Epilepsy

**DOI:** 10.3389/fnhum.2021.770678

**Published:** 2022-01-05

**Authors:** Liping Pan, Yakun Wu, Jie Bao, Dandan Guo, Xin Zhang, Jiajing Wang, Meili Deng, Peiran Yu, Gaoxu Wei, Lulin Zhang, Xiao Qin, Yijun Song

**Affiliations:** ^1^General Medicine Department, Tianjin Medical University General Hospital, Tianjin, China; ^2^Department of Neurology, Tianjin Medical University General Hospital, Tianjin, China; ^3^Department of Neurology, Tangshan Gongren Hospital, Tangshan, China; ^4^Department of Rehabilitation Medicine, Tianjin Medical University General Hospital, Tianjin, China; ^5^School of Basic Medical Science, Tianjin Medical University, Tianjin, China; ^6^Department of Neurology, The First Affiliated Hospital of Fujian Medical University, Fuzhou, China; ^7^Key Laboratory of Central Nerve Injury Repair and Regeneration, Ministry of Education, Tianjin Neurological Institute, Tianjin, China

**Keywords:** working memory, encoding, temporal lobe epilepsy, theta, neural network

## Abstract

**Objective:** The aim of the current study was to investigate the alterations in the neural networks of patients with temporal lobe epilepsy (TLE) during working memory (WM) encoding.

**Methods:** Patients with TLE (*n* = 52) and healthy volunteers (*n* = 35) completed a WM task, during which 34-channel electroencephalogram signals were recorded. The neural networks during WM encoding were calculated in TLE patients with (TLE-WM) and without (TLE-N) WM deficits.

**Results:** Functional connectivity strength decreased, and the theta network was altered in the TLE-WM group, although no significant differences in clinical features were observed between the TLE-N and TLE-WM groups.

**Conclusions:** Not all patients with TLE present with cognitive impairments and alterations in the theta network were identified in TLE patients with functional cognitive deficits.

**Significance:** The theta network may represent a sensitive measure of cognitive impairment and could predict cognitive outcomes among patients with TLE.

## Introduction

Working memory (WM) is a core cognitive function that is vital for higher-level cognition. Information is encoded, stored, and kept available over a short delay during WM to guide future behavior (Barak and Tsodyks, 2014; Wasmuht et al., 2018). Higher-level cognition is impaired in cases of WM deficits, affecting normal functioning in daily activities. WM processes, including the encoding, maintenance, and retrieval phases, are well-known to be associated with distributed network properties and interactions across multiple brain regions (Soreq et al., 2019), and alterations in neural oscillations can occur during any phase of WM but are most prominent during the encoding phase in patients with temporal lobe epilepsy (TLE; Pan et al., 2021). The aim of the current study was to investigate alterations in the neural networks of patients with TLE during WM encoding.

Recent studies examining WM circuits have suggested that a variety of brain regions are involved in WM processing, including the prefrontal and occipital regions, parietal and temporal association cortex, cingulate and limbic areas, and subcortical structures, such as the mediodorsal thalamus and the basal ganglia (Cabeza and Nyberg, 2000; Constantinidis and Procyk, 2004; Constantinidis and Wang, 2004; Linden, 2007; Rottschy et al., 2012; Nee and D'Esposito, 2018). During WM encoding, sustained neuronal activity in the left dorsolateral prefrontal cortex, bilateral occipital, and temporal areas have been reported to contribute to the encoding of location and item features (Heinrichs-Graham and Wilson, 2015; Leavitt et al., 2017). Neurons in the prefrontal cortex (PFC), which play an important role in characterizing WM task-related contents and rules, undergo a succession of rapid state transitions according to task relevance during the encoding phase, modulating other brain areas, and reconfiguring the stimulus-response mapping to guide future behaviors (Stokes et al., 2013; Heinrichs-Graham and Wilson, 2015; Stokes, 2015).

Neural oscillations are thought to enable the efficient transmission and coding of information during the WM process (Roux and Uhlhaas, 2014). Theta activity, especially in the frontal midline cortex, is increased during WM tasks and changed along with task difficulty, and theta activity decline was associated with impaired performance (Sauseng et al., 2010; Brookes et al., 2011; Hsieh and Ranganath, 2014; Ozelo et al., 2014; Tóth et al., 2014). A recent study showed that theta rhythm desynchronization mediated by transcranial alternating current stimulation (tACS) in the frontoparietal network resulted in impaired performance, whereas increased synchronization was related to improved performance (Alekseichuk et al., 2017), suggesting a pivotal role for theta oscillations in the WM process.

Cognitive dysfunction is considered a potential comorbidity among patients with TLE, involving a variety of domains, such as language, attention, executive function, and WM (Helmstaedter and Kockelmann, 2006; Bell et al., 2011; Allone et al., 2017; Chauvière, 2019). Approximately 50–80% of patients with TLE are reported to present with impairments in at least one cognitive domain, most frequently memory (Helmstaedter et al., 2003; Hermann et al., 2006; Wagner et al., 2009; Bell et al., 2011; Helmstaedter and Witt, 2012). Previous neuropsychology studies provided evidence to support WM impairment in TLE patients (Grippo et al., 1996; Abrahams et al., 1999; Wagner et al., 2009; Black et al., 2010). A functional magnetic resonance imaging (fMRI) study that examined WM performance in 36 patients with epilepsy, including 10 patients with TLE, 13 patients with frontal-temporal lobe epilepsy, and 13 patients with frontal lobe epilepsy, suggested that patients with epilepsy demonstrated impairment in all phases of WM compared with control subjects, concluding that seizures disrupted WM network integrity, regardless of what temporal or other brain regions showed impairments (Vlooswijk et al., 2011).

We speculated that the theta network is altered during WM in patients with TLE. To test this hypothesis, in the current study, we compared theta network during the WM encoding phase between patients with TLE and control subjects. Electroencephalography (EEG) signals were recorded during the delayed match-to-sample task, which can differentiate the encoding phase from the other phases and is one of the most commonly used tools for evaluating WM (Daniel et al., 2016). Our findings provide insights into the neural network alterations underlying WM deficits in TLE and provide a sensitive measure of cognitive impairment in these patients. Theta network analysis may represent a valid means for distinguishing cognitive functional impairments and could predict cognitive outcomes among patients with TLE.

## Subjects and Methods

### Subjects

Patients with TLE (*n* = 52; mean age: 34.50 years; range: 23–50 years) were recruited at the outpatient center of Tianjin Medical University General Hospital. The inclusion criteria were as follows: (i) diagnosed with TLE based on symptomatology, MRI, and video-EEG recordings; (ii) normal or corrected-to-normal vision; (iii) Mini-Mental State Examination score >24; and (iv) voluntarily signed the informed consent form and cooperated with the testing protocol. Exclusion criteria were serious neurologic, mental, or systemic diseases; drug abuse; or alcohol dependence. All patients received anti-seizure medication (ASM) treatment, as either monotherapy or multitherapy, including levetiracetam, lamotrigine, oxcarbazepine, or topiramate. Healthy volunteers (*n* = 35; mean age: 33.54 years; range: 25–50 years) with no history of neurologic or psychiatric disease were recruited as the control (Con) group. The demographic and clinical characteristics of the study subjects are shown in Table 1. All subjects provided written informed consent before participating in the study. The study protocol was approved by the Ethics Committee of the Tianjin Medical University General Hospital.

**Table 1:** Demographic and clinical characteristics of subjects.

	**Control**	**TLE**	**TLE-N**	**TLE-WM**
*N*	35	52	21	31
Age (y)	33.54 ± 7.35	34.50 ± 9.37	31.90 ± 8.10	36.68 ± 9.95
Male/Female	18/17	32/20	13/8	19/12
Education (y)	14.86 ± 3.25	13.83 ± 2.76	15.52 ± 2.04	12.48 ± 2.77
Age of onset (y)	–	24.53 ± 11.07	24.40 ± 8.40	24.63 ± 12.93
Disease duration (y)	–	8.56 ± 5.57	7.88 ± 8.12	10.68 ± 10.92
Seizure frequency (per month)	–	2.15 ± 2.32	1.58 ± 2.53	4.65 ± 7.30
AEDs species	–	2.10 ± 0.74	1.95 ± 0.51	2.20 ± 0.85

*Values are mean and standard deviation*.

### Task Procedures and Behavior Performance Phenotyping

**Figure 1:**
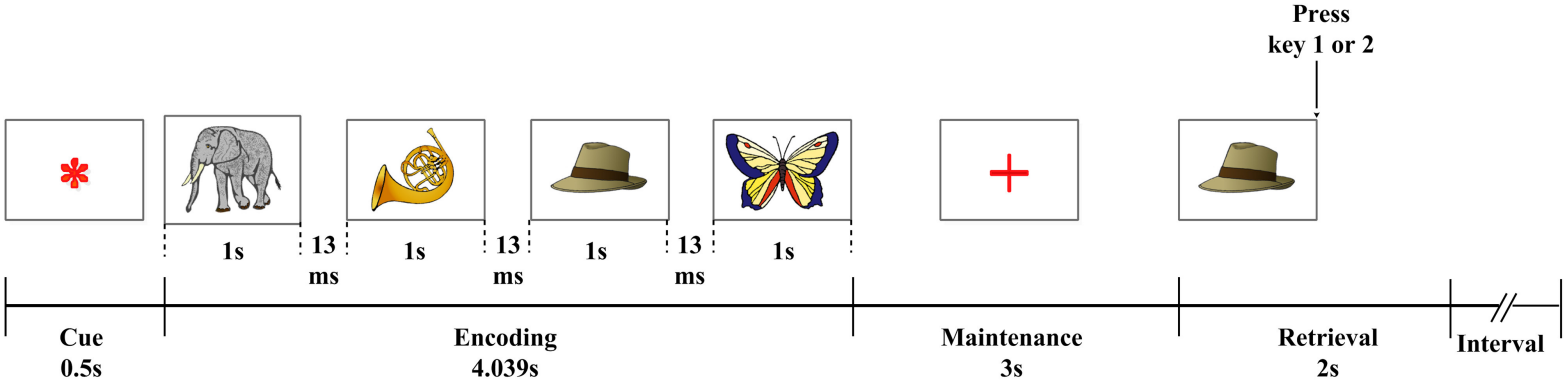
Visual WM paradigm. An asterisk (*), serving as a fixation point, was shown for 0.5 s; four pictures from a memory set were then sequentially presented for 1 s each, separated by an interval of 0.013 s. This was followed by a 3-s maintenance phase, after which a probe image was shown to the subject. Then there was 2 s for the subject to decide whether the probe image was presented among the initial four images from the memory set (Pan et al., 2021).

A visual WM task (Figure 1) was used in the present study. All participants were trained before the test began to ensure that they understood the task procedures. Pictures of objects from the Snodgrass picture set (Rossion and Pourtois, 2004) were presented on a white background. At the beginning of each test, a fixation asterisk (^*^) as a cue was shown in the center of the screen. The asterisk disappeared after 0.5 s, and the encoding phase began; four pictures were presented sequentially on the screen for 1 s each, with the interval between two pictures set to 0.013 s. Each picture was randomly selected from the picture set. The encoding phase was followed by a 3-s maintenance phase and then the retrieval phase. During the retrieval phase, a probe image appeared on the screen, and subjects were given 2 s to select whether the probe image matched one of the pictures presented during the encoding phase. If the answer was yes, the subjects pressed button 1 on the keyboard; otherwise, they would push button 2. The trial was over after the subjects push the button or after 2 s the probe image presented if the subjects did not push any button, and after 4 s the next trial began. Participants were required to complete six blocks of the task, each of which contained 10 trials. Task performance was measured in terms of the mean reaction time (RT) for correct trials and response accuracy (ACC).

Raw RT and ACC values for all patients were converted into z-scores based on the mean of the Con group, with 1.5 SD of the Con group defined as the normal range of WM behavior performance, and all responses beyond 1.5 SD classified as impaired performance (Reyes et al., 2019). The TLE group was divided into two subgroups according to the behavior performance phenotype: the TLE-WM group, which including patients with TLE for whom either the RT or ACC values were beyond the normal range (performance-impaired), and the TLE-N group, which including patients with TLE for whom the performance was within the normal range (performance-normal).

### EEG Recording and Data Preprocessing

EEG signals were recorded from 34 channels arrayed over the scalp using a NicoletOne EEG system (Natus Medical, Pleasanton, CA, USA), with a sampling rate of 1,024 Hz and impedance maintained below 5 kΩ. The scalp electrodes of channels 1–34 (Fp1, Fp2, F7, F3, Fz, F4, F8, FT7, FC3, FCz, FC4, FT8, T7, C3, Cz, C4, T8, TP7, CP3, CPz, CP4, TP8, P7, P3, Pz, P4, P8, PO3, PO4, O1, Oz, O2, AF3, and AF4) were positioned according to an extended version of the international 10–20 system. The reference electrode was located near Cz, and the ground electrode was located on the forehead. EEG signals were low-pass–filtered (100 Hz), notch-filtered (49–51 Hz), and re-referenced to a common average reference value. Baseline drift was removed in MATLAB v2012a (MathWorks, Natick, MA, USA), and eye movement and myoelectricity artifacts were removed with the covariance method and blind source separation using the EEGLAB toolbox (Delorme and Makeig, 2004).

### Analysis of Neural Oscillation Patterns During WM Encoding

The power spectral density (PSD) of delta (0.05–4 Hz), theta (4–8 Hz), alpha (8–13 Hz), beta (13–30 Hz), and gamma (30–100 Hz) frequency bands were calculated with the application of a short-time Fourier transform (STFT) during WM encoding, using a 0.4 s–wide Hamming window and 0.5-Hz frequency smoothing (Zhang et al., 2016). The STFT formula was as follows:


(1) $$STFT\left(f,t\right)=\int_{-\infty }^{+\infty }[x(t)g(t-\tau )e^{-j2\pi f\tau }d\tau$$

The variable *x(t)* was set as the EEG signal, and *g(t)* was set as a window function. The PSD values of the delta, theta, alpha, beta, and gamma bands were compared, and the frequency band with maximum PSD was defined as the prominent frequency band and retained for subsequent analysis.

The PSD of prominent frequency band under resting conditions (i.e., 5 min with eyes closed before the WM task began) was calculated as the baseline. The PSD of WM efficiency was defined as follows:


(2) $$\begin{array}{l} P_{raw}-P_{rs}=P_{wm\;} \end{array}$$

where *P*_*raw*_ is the PSD calculated during the WM task; *P*_*rs*_ is the baseline PSD; and *P*_*wm*_ is the PSD of WM efficiency, which reflects the change in EEG power during the WM process and was compared among groups.

To identify the prominent brain region(s) involved in WM encoding, the EEG channels were grouped into four clusters: the frontal region (F, including Fp1, Fp2, F3, Fz, F4, FC3, FCz, FC4, AF3, and AF4); the central-parietal region (C, including C3, Cz, C4, CP3, CPz, CP4, P3, Pz, and P4); the occipital region (O, including PO3, PO4, O1, Oz, and O2); and the temporal region (T, including FT7, FT8, T3, T4, TP7, TP8, T5, and T6). The PSD of the prominent frequency band in each brain region was calculated, and the region(s) with the highest PSD during the WM task was defined as the prominent brain region(s).

### Analysis of Neural Network Patterns During WM Encoding

To analyze the neural network patterns during WM encoding, the directional transfer function (DTF), which is based on the concept of Granger causality and obtained from the framework of the multivariate autoregressive (MVAR) model (Kaminski et al., 2001; Babiloni et al., 2005), was used to evaluate the functional connectivity strengths among EEG channels and different brain regions. The DTF from channel *j* to channel *i* represents the causal influence from channel *j* to channel *i* at frequency *f* and was defined as:


(3) $$\begin{array}{l} \gamma_{ij}\left(f\right)=\frac{|H\left(f\right){|}^{2}}{\sum_{m=1}^{k}|Him\left(f\right){|}^{2}} \end{array}$$

Where γ_*ij*_*(f)* represents the ratio between influence from channel *j* to channel *i* and the joint influences from all the other channels to channel *i*; *k* represents the channel numbers; and *H* represents the transfer matrix of the system (Kaminski et al., 2001; Seth, 2010).

The 34 channels denoted different network nodes that formed the causality network. The global brain functional connectivity (*DTF*_*g*_) was defined as the mean value of all elements in the DTF matrix, which directly reflects the functional connectivity strength of the encoding network associated with WM. The formula for *DTF*_*g*_ was as follows:


(4) $$\begin{array}{l} DTF_{g}=\frac{\sum_{i\in \text{K}}\sum_{j\neq i\in K}DTFij}{n\left(n-1\right)} \end{array}$$

where *n* represents the number of channels, and K represents the set of channels in the network. The *DTF*_*g*_ values during the WM encoding phase were calculated for the two TLE subgroups and healthy controls, and comparisons among groups were conducted.

The connectivity strength of channel *i* (*DTF*_*i*_) was defined as the functional connectivity in the network associated with channel *i*, including the average connectivity from other channels to channel *i* and from channel *i* to other channels in the network. The *DTF*_*i*_ was calculated as follows:


(5) $$\begin{array}{l} DTF_{i}=\frac{1}{2\left(n-1\right)}\underset{j\neq i\in G}{\sum }\left(DTF_{ji}+DTF_{ij}\right) \end{array}$$

where *n* is the number of channels, and *G* is the set of channels in the DTF matrix. *DTF*_*i*_ is an important measure of the “activity” of each channel across the network. The spatial distribution maps during WM encoding were drawn based on the *DTF*_*i*_ for each channel, and the most prominent channel during the WM encoding phase was identified.

To further analyze the spatial distribution pattern of the function network during WM encoding, the connectivity strength across brain regions was calculated. The connectivity strength from brain region *l* to brain region *k* (*DTF*_*kl*_) was defined as follows:


(6) $$\begin{array}{l} DTF_{kl}=\frac{1}{mn}\underset{m\in K}{\sum }\underset{n\in L}{\sum }DTF_{ij} \end{array}$$

Where *m* and *n* are the number of channels in brain regions *k* and *l*, respectively; and *K* and *L* are the set of channels in brain regions *k* and *l*, respectively. Comparisons of the functional network patterns during the WM encoding phase were performed among the TLE-N group, the TLE-WM group, and healthy controls, and characteristic changes during the WM encoding phase were identified in patients with TLE.

### Statistical Analysis

SPSS v20.0 (SPSS Inc., Chicago, IL, USA) was used for all statistical analyses, and the data are expressed as the mean ± the standard error of the mean (unless otherwise noted). For all the statistical tests, the level of significance was set to α = 0.05 (*p* < 0.05). In this article, data normality was confirmed with the Shapiro-Wilk test (*P* > 0.05), the *t*-test was used to assess the significance of differences between two groups, and Levene's test was used to assess the homogeneity of error variances across groups. The Chi-square test was used to analyze dichotomous variables. Comparisons among more than three groups were performed using one-way analysis of variance, and *post-hoc* analyses were performed using the least significant difference test. Pearson's correlation analysis was performed to evaluate the relationship between EEG network activity during WM encoding and performance.

## Results

### Demographic and Clinical Characteristics and Performance of Subjects

The demographic and clinical characteristics of all subjects are shown in Table 1. No significant differences were identified between the TLE group and the Con group for age (*t* = 0.499, *p* = 0.619), sex distribution (*t* = 1.430, *p* = 0.232), or education (*t* = 0.449, *p* = 0.116). The TLE group was divided into two subgroups: the TLE-N group and the TLE-WM group. No significant differences in age (*F* = 1.965, *df* = 2, *p* = 0.147) or sex distribution (*F* = 1.432, *df* = 2, *p* = 0.489) were observed among the TLE-N group, the TLE-WM group, and the Con group. For education level, a significant difference was observed among the TLE-WM group, the TLE-N group, and the Con group (*F* = 8.957, *df* = 2, *p* < 0.001). The TLE-WM group was less educated than the Con group or the TLE-N group (Con. vs. TLE-WM, LSD-t = 3.404, *p* = 0.006; TLE-N vs. TLE-WM, LSD-t = 3.805, *p* < 0.001). No significant difference in education level was observed between the TLE-N group and the Con group (LSD-t = 0.854, *p* = 0.395). No significant differences between the TLE-N group and the TLE-WM group were identified for age of TLE onset (*t* = 0.069, *p* = 0.945), disease duration (*t* = 0.971, *p* = 0.336), seizure frequency (*t* = 1.808, *p* = 0.077), and or the number of anti-seizure medications (ASM) used (*t* = 1.301, *p* = 0.200).

**Figure 2:**
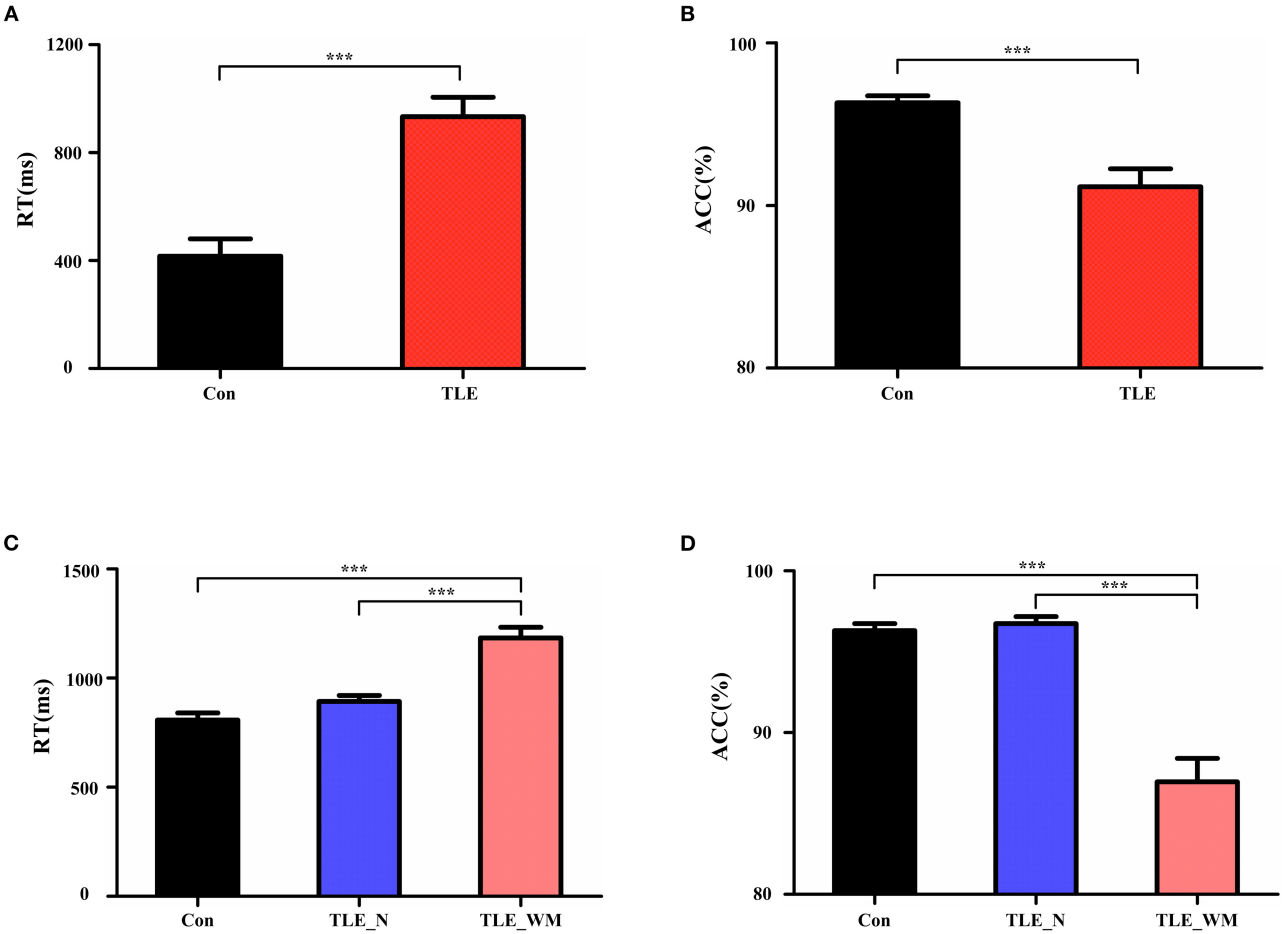
Comparisons of task performance. **(A,B)** Comparisons of RT and ACC results between the TLE group and the Con group. **(C,D)** Comparisons of RT and ACC results between the TLE-N group, the TLE-WM group, and the Con group. ***p < 0.001.

Comparisons of memory test performance among groups are shown in Figure 2. The mean RT was longer in the TLE group than in the Con group (*t* = 5.339, *p* < 0.001; Figure 2A), and ACC was lower in the TLE group than that in the Con group (*t* = 4.391, *p* < 0.001; Figure 2B). Significant differences were observed among the TLE-N group, the TLE-WM group, and the Con group for both RT (*F* = 28.174, *df* = 2, *p* < 0.001; Figure 2C) and ACC (*F* = 36.322, *df* = 2, *p* < 0.001; Figure 2D). Compared with the TLE-N group and the Con group, the RT was longer (Con vs. TLE-WM, LSD-t = 7.299, *p* < 0.001; TLE-N vs. TLE-WM, LSD-t = 4.931, *p* < 0.001), and the ACC was lower (Con vs. TLE-WM, LSD-t = 7.642, *p* < 0.001; TLE-N vs. TLE-WM, LSD-t = 7.023, *p* < 0.001) in the TLE-WM group. No significances between the TLE-N group and the Con group were observed for RT (LSD-t = 2.005, *p* = 0.141) or ACC (LSD-t = 0.720, *p* = 0.852).

### The Prominent Frequency Band During the WM Encoding Phase

**Figure 3:**
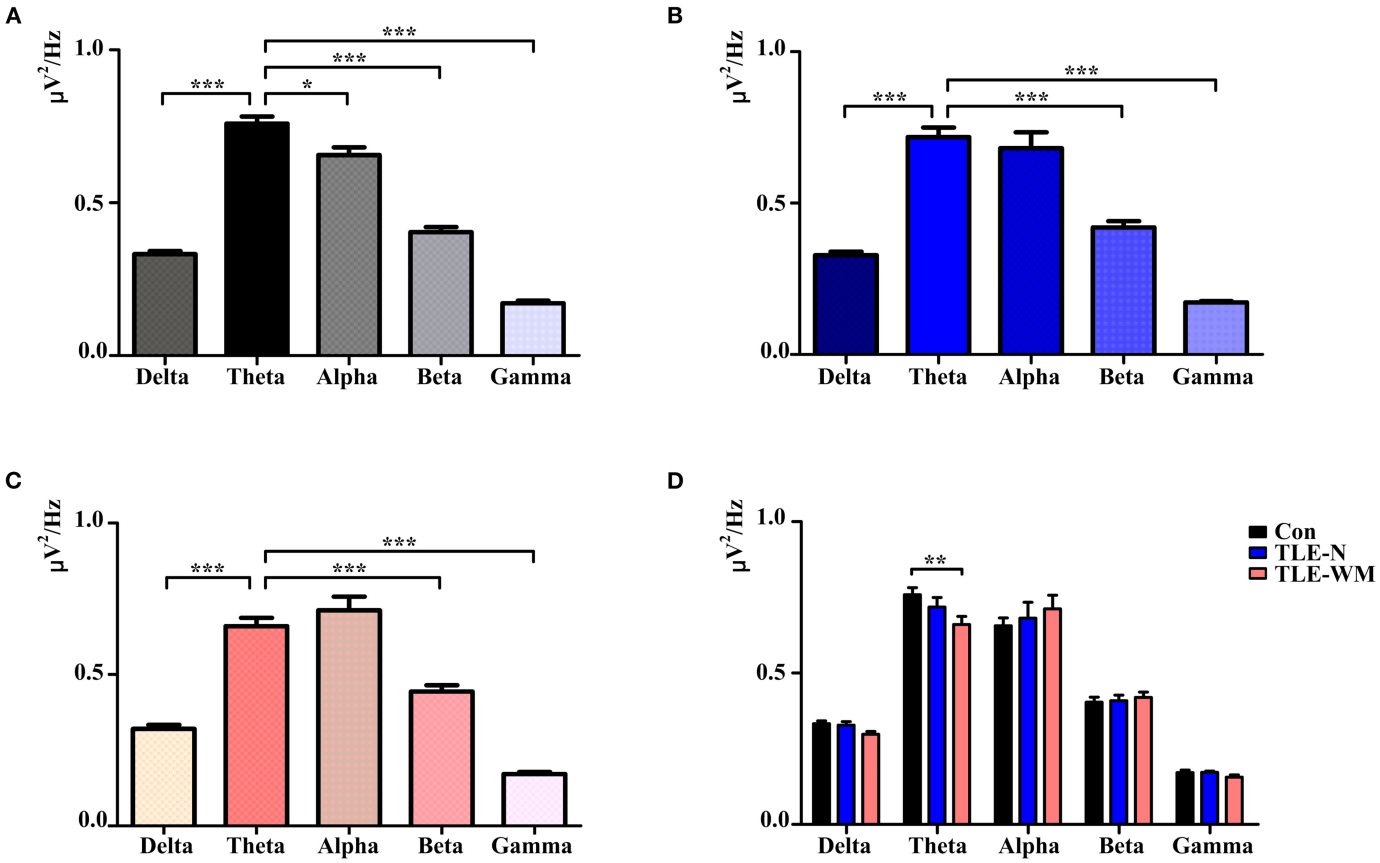
Neural oscillations during the WM encoding phase across the three groups. Comparisons of PSD values among the delta, theta, alpha, beta, and gamma frequencies in the Con group **(A)**, TLE-N group **(B)**, and TLE-WM group **(C)**. **(D)** Comparisons of PSD values associated with different frequency bands among the three groups. *p < 0.05, **p < 0.01, and ***p < 0.001.

To describe the characteristic pattern of neural oscillations that occur during the WM encoding phase, we compared the average PSD of 34 channels within the delta, theta, alpha, beta, and gamma frequency bands. As shown in Figure 3A, in the Con group, significant differences were observed among the average PSD values of the five frequency bands (*F* = 176.425, *df* = 4, *p* < 0.001), and the PSD value of the theta frequency band was larger than the PSD values of other frequency bands (theta vs. delta: LSD-t = 16.115, *p* < 0.001; theta vs. alpha: LSD-t = 2.996, *p* = 003; theta vs. beta: LSD-t = 12.963, *p* < 0.001; and theta vs. gamma: LSD-t = 22.738, *p* < 0.001). In the TLE-N group, significant differences were observed among the average PSD values of the five frequency bands (*F* = 56.749, *df* = 4, *p* < 0.001), and the average PSD value of the theta frequency band was larger than those of the delta, beta, and gamma bands (theta vs. delta: LSD-t = 9.206, *p* < 0.001; theta vs. beta: LSD-t = 6.949, *p* < 0.001; and theta vs. gamma: LSD-t = 14.327, *p* < 0.001), whereas no significant difference was observed between the average PSD values of the theta and alpha bands (LSD-t = 0.857, *p* = 0.394; Figure 3B). In the TLE-WM group, significant differences were observed among the average PSD values of the five frequency bands (*F* = 53.639, *df* = 4, *p* < 0.001), and the average PSD value of the theta frequency band was significantly larger than those of the delta, beta, and gamma frequency bands (theta vs. delta: LSD-t = 8.227, *p* < 0.001; theta vs. beta: LSD-t = 5.336, *p* < 0.001; and theta vs. gamma: LSD-t = 9.213, *p* < 0.001), whereas no significant difference was observed between the average PSD values of the theta and alpha frequency bands (LSD-t = 1.209, *p* = 0.201; Figure 3C).

When the PSD values were compared across the three groups, significant differences were observed between the TLE-N group, the TLE-WM group, and the Con group for the average PSD value of the theta frequency band (*F* = 4.893, *df* = 2, *p* = 0.011), and the average PSD value of the theta frequency band in the TLE-WM group was significantly smaller than that in the Con group (LSD-t = 3.114, *p* = 0.003). No significant differences across the three groups were observed for the average PSD values of the delta, alpha, beta, or gamma frequency bands (delta: *F* = 2.896, *df* = 2, *p* = 0.062; alpha: *F* = 0.249, *df* = 2, *p* = 0.781;beta: *F* = 0.223, *df* = 2, *p* = 0.801;gamma: *F* = 1.068, *df* = 2, *p* = 0.353; Figure 3D). Therefore, the theta frequency band was considered to represent the prominent frequency band during the WM encoding phase, and subsequent analyses were focused to this frequency band.

### Characteristic Spatial Distribution Pattern of Neural Oscillations During the WM Encoding Phase

**Figure 4:**
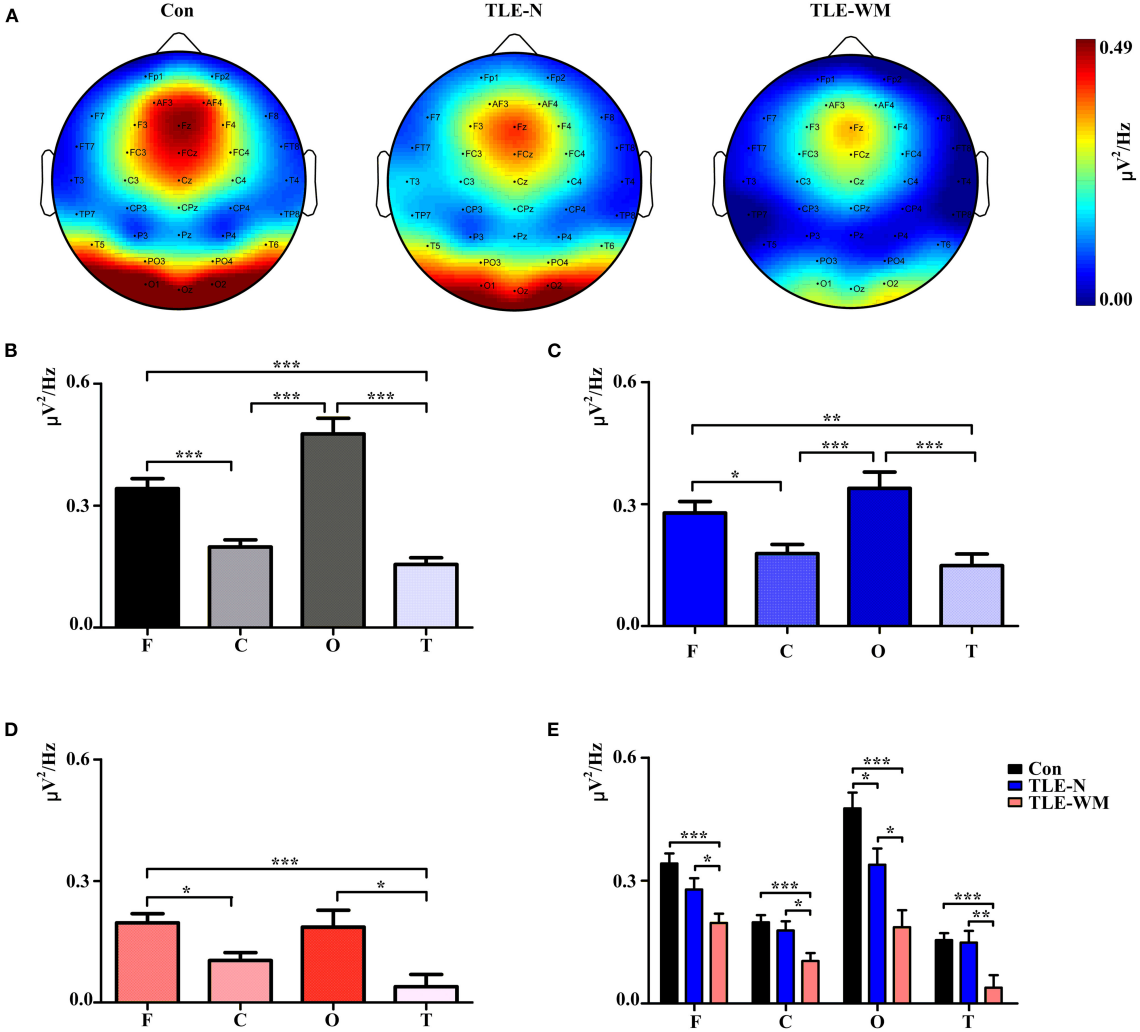
Spectral distribution and histogram of theta power during the WM encoding phase in the three groups. **(A)** Spectral distribution of theta power in the three groups. Power is indicated by color. Histogram of theta power in different brain regions for the Con group **(B)**, the TLE-N group **(C)**, and the TLE-WM group **(D)**. **(E)** Comparisons of theta power associated with different brain regions across the three groups. C, central-parietal region; F, frontal region; T, temporal region; O, occipital region. *p < 0.05, **p < 0.01, and ***p < 0.001.

The topographic maps generated for the TLE-N group, the TLE-WM group, and the Con group during the WM encoding phase are shown in Figure 4A, which indicated that the theta *P*_*wm*_ was primarily concentrated in the frontal and occipital regions. In the Con group, significant differences were observed for the theta *P*_*wm*_ values measured among the frontal, parietal, occipital, and temporal regions (*F* = 32.504, *df* = 3, *p* < 0.001). The theta *P*_*wm*_ value for the frontal region measured as significantly larger than those in the parietal (LSD-t = 4.771, *p* < 0.001) and temporal regions (LSD-t = 6.292, *p* < 0.001), and the theta *P*_*wm*_ value measured in the occipital region was also significantly larger than those in the parietal (LSD-t = 6.400, *p* < 0.001) and temporal regions (LSD-t = 7.563, *p* < 0.001), with no significant difference observed between theta *P*_*wm*_ values between the frontal and occipital regions (LSD-t = 2.505, *p* = 0.087; Figure 4B). In the TLE-N group, significant differences in the theta *P*_*wm*_ values were observed among the frontal, parietal, occipital, and temporal regions (*F* = 8.514, *df* = 3, *p* < 0.001), revealing the theta *P*_*wm*_ values for the frontal and occipital regions were significantly larger than those for the parietal (frontal vs. parietal, LSD-t = 2.304, *p* = 0.024; occipital vs. parietal, LSD-t = 3.693, *p* < 0.001) and temporal regions (frontal vs. temporal, LSD-t = 3.057, *p* = 0.003; occipital vs. temporal, LSD-t = 4.481, *p* < 0.001). No significant difference was observed for the theta *P*_*wm*_ values between the frontal and occipital regions (LSD-t = −1.407, *p* = 0.164; Figure 4C). Comparisons of the theta *P*_*wm*_ values among different brain regions in the TLE-WM group are shown in Figure 4D, revealing significant differences (*F* = 6.486, *df* = 3, *p* < 0.001). The theta *P*_*wm*_ value in the frontal region was higher than that in the parietal (LSD-t = 3.124, *p* = 0.016) and temporal regions (LSD-t = 4.200, *p* = 0.001), and the theta *P*_*wm*_ value in the occipital region was higher than that in the temporal region (LSD-t = 2.862, *p* = 0.035). No significant difference in the theta *P*_*wm*_ values between the frontal and occipital region was observed (LSD-t = 0.254, *p* = 0.800) in the TLE-WM group.

Comparisons of the theta *P*_*wm*_ values across different brain regions and among groups are shown in Figure 4E. Significant differences among groups were observed for the frontal (*F* = 9.847, *df* = 2, *p* < 0.001), parietal (*F* = 7.184, *df* = 2, *p* = 0.001), occipital (*F* = 12.702, *df* = 2, *p* < 0.001), and temporal (*F* = 7.113, *df* = 2, *p* = 0.001) regions. Compared with the Con group, the theta *P*_*wm*_ values in the frontal, parietal, occipital, and temporal regions of the TLE-WM group were significantly lower (frontal, LSD-t = 4.433, *p* < 0.001; parietal, LSD-t = 3.657, *p* < 0.001; occipital, LSD-t = 5.030, *p* < 0.001; temporal, LSD-t = 3.477, *p* = 0.001), and the theta *P*_*wm*_ in each region was also lower in the TLE-WM group than those in the TLE-N group (frontal, LSD-t = 2.179, *p* = 0.032; parietal, LSD-t = 2.503, *p* = 0.014; occipital, LSD-t = 2.598, *p* = 0.011; temporal, LSD-t = 2.896, *p* = 0.005).

### Network Alterations Associated With Impaired WM in Patients With TLE

#### Functional Connectivity Strength During the WM Encoding Phase

**Figure 5:**
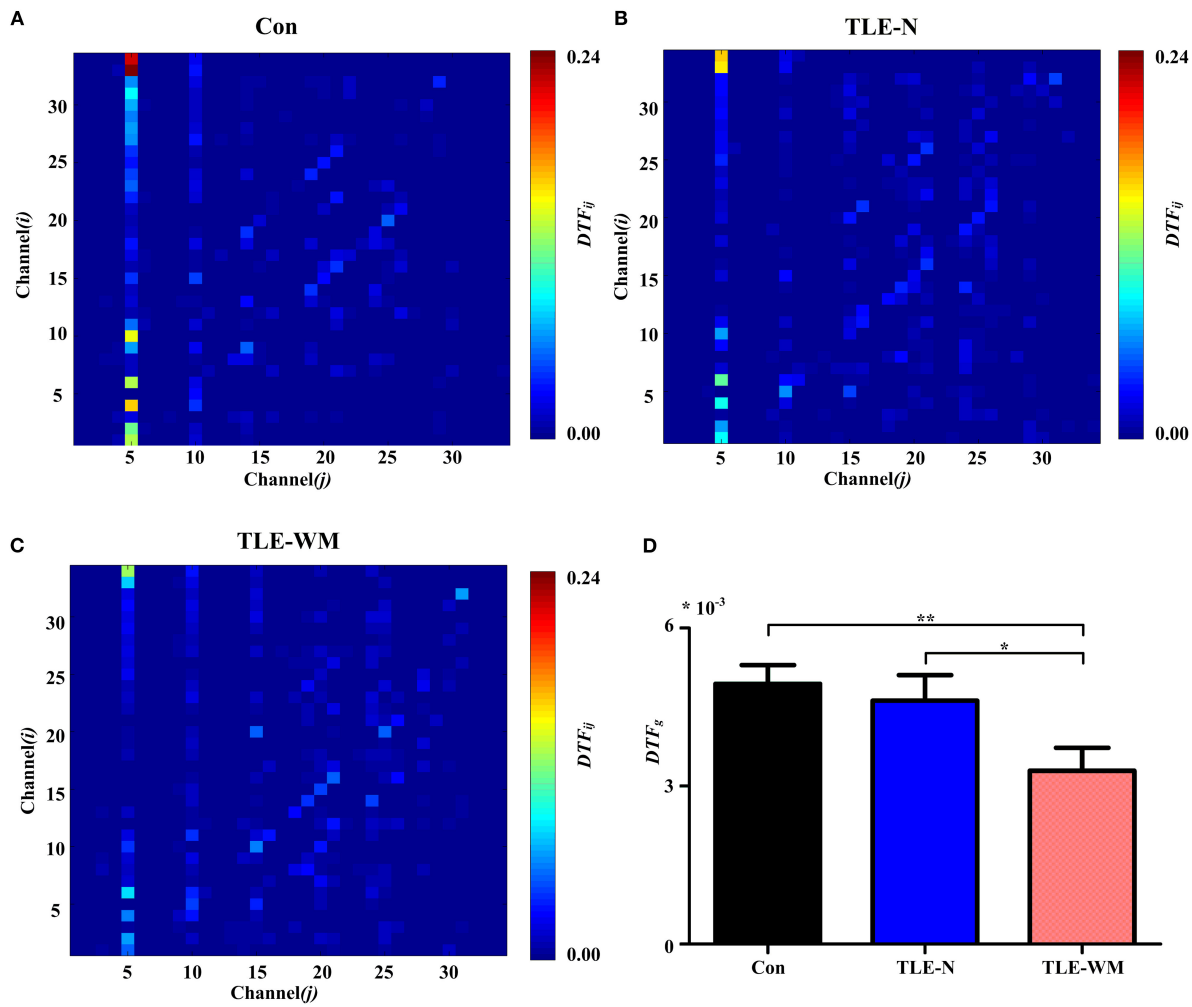
Functional connectivity across channels during the WM encoding phase. Connectivity matrices of EEGs in the Con group **(A)**, TLE-N group **(B)**, and TLE-WM group **(C)**. Nodes 1–34 represent Channels 1–34 (i.e., Fp1, Fp2, F7, F3, Fz, F4, F8, FT7, FC3, FCz, FC4, FT8, T7, C3, Cz, C4, T8, TP7, CP3, CPz, CP4, TP8, P7, P3, Pz, P4, P8, PO3, PO4, O1, Oz, O2, AF3, and AF4, respectively). The connectivity strengths from channel *j* to channel *i* are represented by the different colors. The connectivity strength in the Fz channel (channel 5) was strong. **(D)** Comparisons of the average DTF values for 34 channels among the three groups. The average connectivity strength in the TLE-WM group decreased compared with those of the Con group and the TLE-N group. *p < 0.05 and **p < 0.01.

The DTF method was used to estimate the functional connectivity of the theta frequency band among electrodes during the WM encoding phase in the TLE-N group, the TLE-WM group, and the Con group, and the DTF matrices in the Con group, the TLE-N group, and the TLE-WM group were shown in Figures 5A–C, respectively. The DTF was primarily concentrated in the frontal region, especially in the Fz channel. A significant difference in the *DTF*_*g*_ values was observed among the TLE-N group, the TLE-WM group, and the Con group (*F* = 4.587, *df* = 2, *p* = 0.013). Compared with the Con and TLE-N groups, the *DTF*_*g*_ value was lower in the TLE-WM group (Con vs. TLE-WM, LSD-t = 2.914, *p* = 0.005; TLE-N vs. TLE-WM, LSD-t = 2.133, *p* = 0.036). No significant difference was observed for the *DTF*_*g*_ values between the TLE-N group and the Con group (LSD-t = 0.519, *p* = 0.606; Figure 5D).

#### The Functional Connectivity Distribution During the WM Encoding Phase

**Figure 6:**
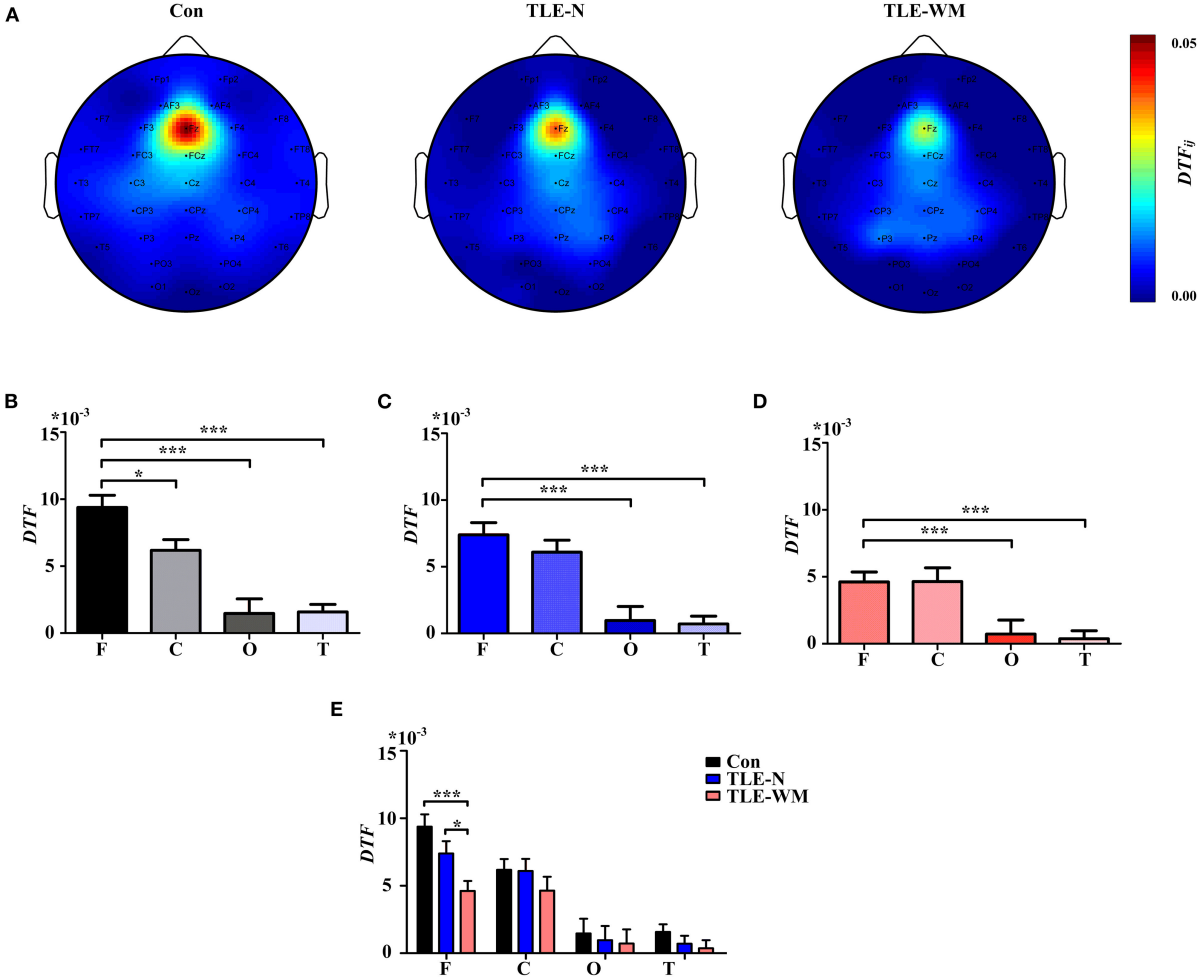
Spectral distribution and histogram showing functional connectivity strength in the theta frequency band during the WM encoding phase across the three groups. **(A)** Spectral distribution of functional connectivity strength across the three groups. Connectivity strength is indicated by color. Histogram of connectivity strength in the theta frequency band associated with different brain regions in the Con group **(B)**, the TLE-N group **(C)**, and the TLE-WM group **(D)**. Connectivity strength in the frontal region was prominent. **(E)** Comparison of connectivity strength in the theta frequency band associated with different brain regions across the three groups. Connectivity strength in the frontal region of the TLE-WM group was weak. *p < 0.05 and ***p < 0.001.

The functional connectivity distribution during the WM encoding phase was explored among the three groups (Figure 6A). In the Con group, significant differences in *DTF* values were observed among the frontal, parietal, occipital, and temporal regions (*F* = 21.320, *df* = 3, *p* < 0.001), and the *DTF* value in the frontal region was significantly larger than those in other regions (frontal vs. parietal, LSD-t = 2.585, *p* = 0.011; frontal vs. occipital, LSD-t = 6.597, *p* < 0.001; frontal vs. temporal, LSD-t = 6.814, *p* < 0.001; Figure 6B). In the TLE-N group, significant differences in *DTF* values were observed among the brain regions (*F* = 15.867, *df* = 3, *p* < 0.001), and the *DTF* value in the frontal region was significantly larger than those in the occipital and the temporal regions (frontal vs. occipital, LSD-t = 5.236, *p* < 0.001; frontal vs. temporal, LSD-t = 5.583, *p* < 0.001), whereas no significant difference in *DTF* values was observed between the frontal and parietal regions (LSD-t = 1.005, *p* = 0.318; Figure 6C). In the TLE-WM group, significant differences in *DTF* values were observed among different brain regions (*F* = 8.141, *df* = 3, *p* < 0.001). The *DTF* value in the frontal region was significantly larger than those in the occipital (LSD-t = 3.422, *p* = 0.001) and temporal regions (LSD-t = 3.943, *p* < 0.001). No significant difference in *DTF* values was observed between the frontal and parietal regions (LSD-t = 0.027, *p* = 0.978; Figure 6D). Therefore, the frontal region was considered to be the prominent region of brain network activity during the WM encoding phase.

Comparisons of the *DTF* values for different regions were examined among the Con, TLE-N, and TLE-WM groups, as shown in Figure 6E. Significant differences in the *DTF*_*frontal*_ values were observed among groups (*F* = 8.600, *df* = 2, *p* < 0.001). No significant differences were observed among the control, TLE-N, and TLE-WM groups for the DTF values of the parietal (*F* = 0.870, *df* = 2, *p* = 0.425), occipital (*F* = 0.137, *df* = 2, *p* = 0.872), or temporal (*F* = 1.249, *df* = 2, *p* = 0.292) regions. Compared with those for the Con group and the TLE-N group, the *DTF*_*frontal*_ value for the TLE-WM group was significantly lower (Con vs. TLE-WM, LSD-t = 4.139, *p* < 0.001; TLE-N vs. TLE-WM, LSD-t = 2.089, *p* = 0.040).

#### Alterations in the Network Connectivity Between Brain Regions During the WM Encoding Phase

**Figure 7:**
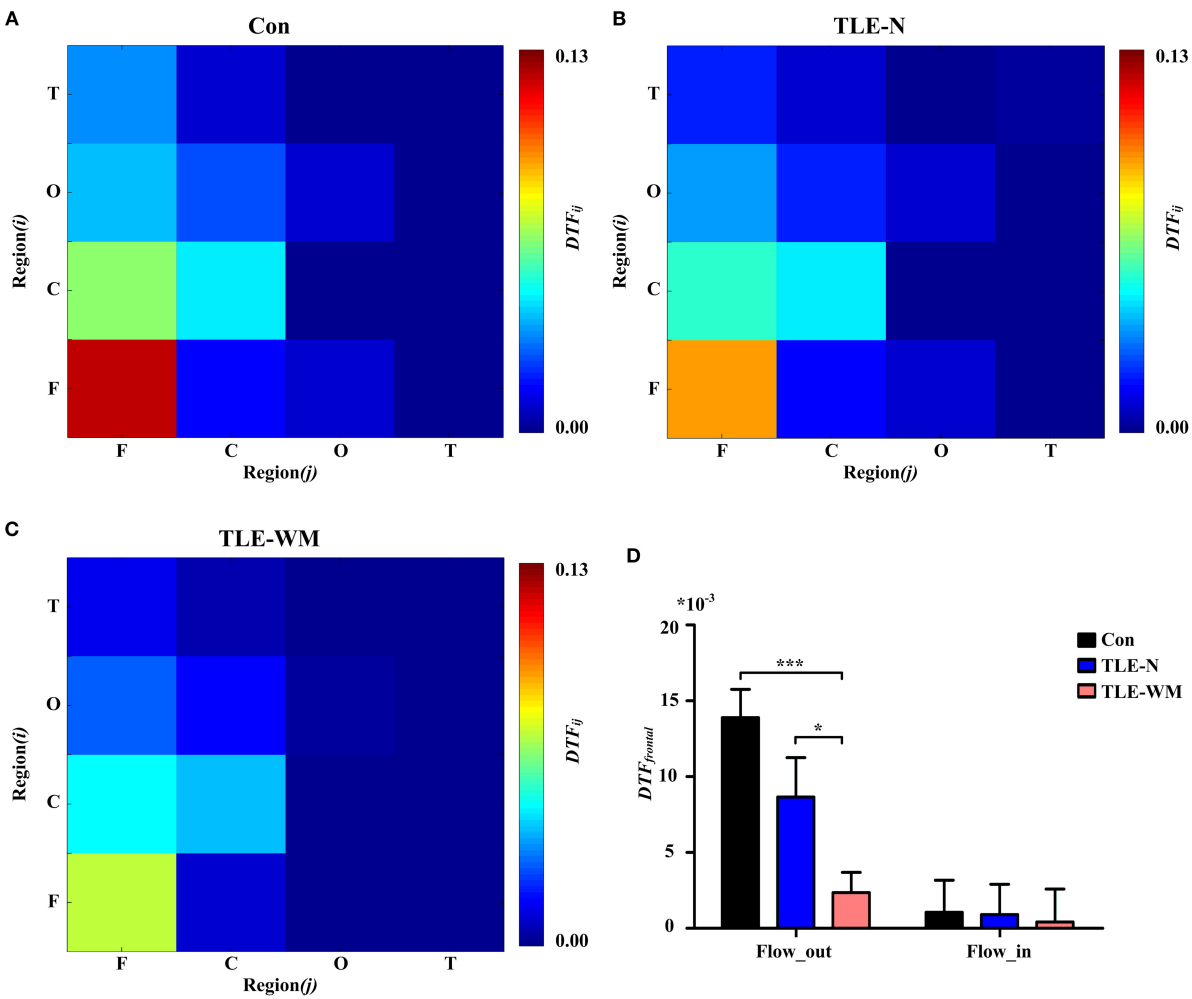
Functional connectivity across brain regions during the WM encoding phase. Connectivity matrices of brain regions in the Con group **(A)**, TLE-N group **(B)**, and TLE-WM group **(C)**. **(D)** Comparison of the average DTF value of the frontal region among the three groups. *p < 0.05 and ***p < 0.001.

To explore the alterations in brain network connectivity observed during the WM encoding phase in more detail, analyses of the *DTF* values from the frontal region to other regions (*DTF*_*out*_) and from other regions to the frontal region (*DTF*_*in*_) were performed. As shown in Figure 7, significant differences in the *DTF*_*out*_ values were observed among the Con, TLE-N, and TLE-WM groups (*F* = 8.600, *df* = 2, *p* < 0.001). The *DTF*_*out*_ value for the TLE-WM group was lower than those for the Con (LSD-t = 5.007, *p* < 0.001) and TLE-N groups (LSD-t = 2.158, *p* = 0.035). No significant differences in *DTF*_*in*_ were observed among the three groups (*F* = 0.026, *df* = 2, *p* = 0.974).

### Correlation Analysis Between Theta Oscillations and Memory Test Performance

**Figure 8:**
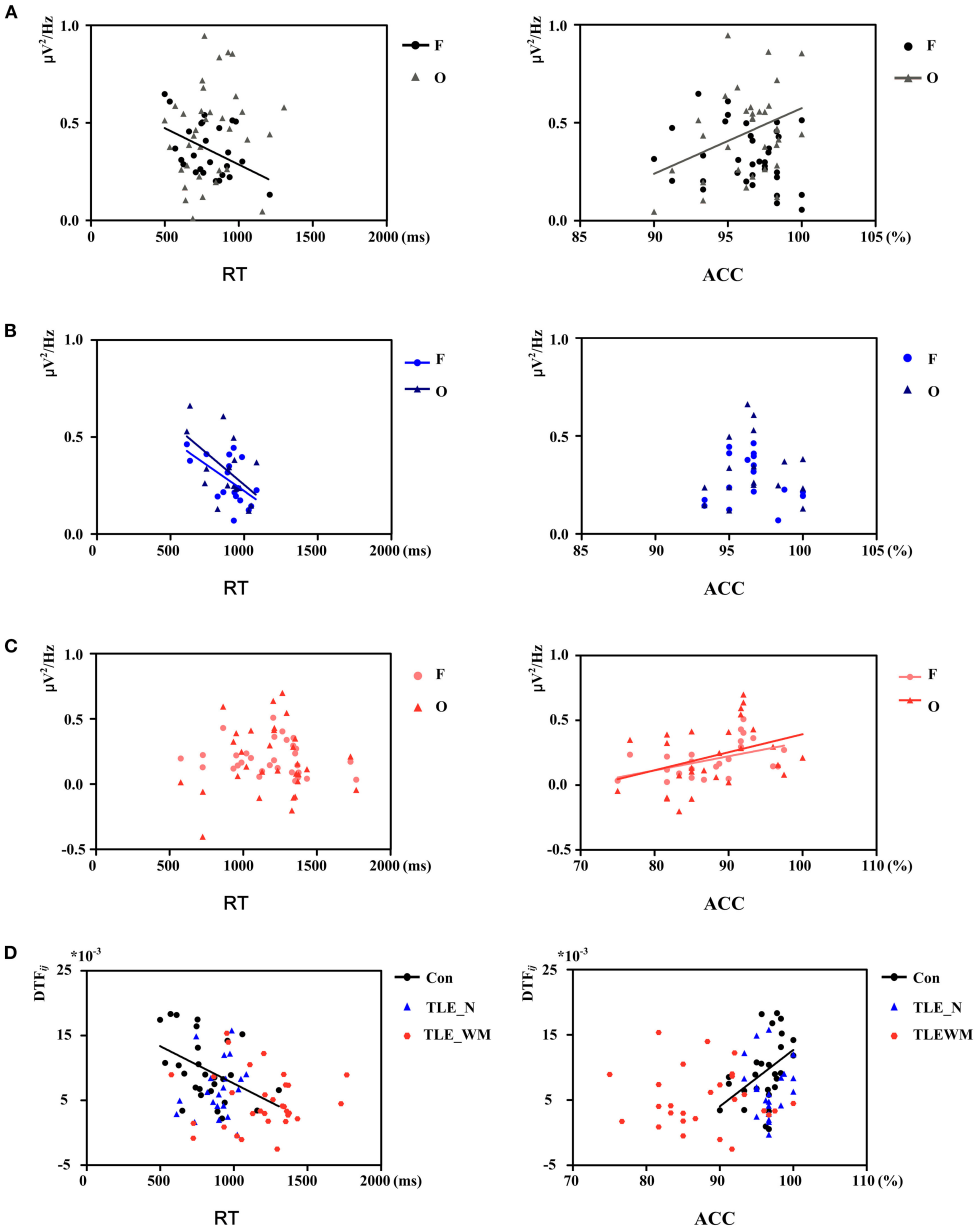
Correlation between theta network activity and performance on the WM task. Correlation between theta *P*_*wm*_ values in different brain regions and performance in the Con group **(A)**, TLE-N group **(B)**, and TLE-WM group **(C)**. **(D)** Correlation between theta DTF values and performance.

To illuminate the relation between the theta oscillations and memory test performance, Pearson' correlation analysis was applied. In the Con group, as shown in Figure 8A, the *P*_*wm*_ value for the frontal region was negatively correlated with mean RT (*r* = −0.441, *p* = 0.024) but was not correlated with ACC (*r* = −0.210, *p* = 0.227). The *P*_*wm*_ value in the occipital region was positively correlated with ACC (*r* = 0.355, *p* = 0.043) but not with RT (*r* = 0.125, *p* = 0.383). In the TLE-N group, a negative correlation was identified between the RT and *P*_*wm*_ values for both the frontal (*r* = −0.565, *p* = 0.015) and occipital regions (*r* = −0.539, *p* = 0.026), and no correlations were identified between the ACC and *P*_*wm*_ values in prominent regions of theta oscillation (frontal: *r* = −0.149, *p* =0.555; occipital: *r* = 0.022, *p* = 0.943; Figure 8B). In the TLE-WM group, a positive correlation was identified between ACC and the *P*_*wm*_ values in the frontal (*r* = 0.482, *p* = 0.015) and occipital (*r* = 0.381, *p* = 0.045) regions, whereas no correlations were observed between the RT and *P*_*wm*_ values in prominent regions of theta oscillation (frontal: *r* = −0.167, *p* = 0.377; occipital: *r* = 0.035, *p* = 0.850; Figure 8C).

Correlations between memory test performance and the function connectivity strength of the frontal region are shown in Figure 8D. *DTF*_*frontal*_ strength was negative correlated with RT (*r* = −0.429, *p* = 0.032) and positively correlated with ACC (*r* = 0.429, *p* = 0.029) in the Con group. whereas in the TLE-N and TLE-WM groups, no correlations were observed between memory test performance and the functional connectivity strength (TLE-N: RT, *r* = 0.193, *p* = 0.401; ACC, *r* = 0.028, *p* = 0.905; TLE-WM: RT, *r* = −0.042, *p* = 0.823; ACC: *r* = 0.115, *p* = 0.537).

## Discussion

In the current study, we compared neural network connectivity in the theta frequency band during the WM encoding phase between patients with TLE and healthy controls by measuring EEG activity and examined the relationships between the theta network and memory performance following a WM task. Our results showed that the theta network was involved in the WM encoding phase, and the frontal region was the most prominent region. Alterations in theta network connectivity were observed in patients with TLE. Stronger theta network activity may be associated with shorter RT and higher ACC.

Theta oscillations play an important role during the WM process (Lisman, 2010; Rutishauser et al., 2010; Sauseng et al., 2010; Polanía et al., 2012; Berger et al., 2019). Our results was accordance with the previous researches. Increased theta activity was observed during the WM encoding phase in a Sternberg-like WM task, as reported by Raghavachari et al. (2001), and theta power was shown to decrease under conditions of WM impairment (Brookes et al., 2011). In our study, theta power was stronger than that of the other frequency bands during the WM encoding phase, and the theta network strength was notably weaker in patients with TLE who demonstrated impaired performance on the WM task, which indicated the important role played by the theta network during the WM encoding phase. Theta oscillations may mirror a gating mechanism that promotes task-relevant information processing and suppresses task-irrelevant information processing (Raghavachari et al., 2001). During higher cognitive processes, in which inter-regional cooperation is indispensable, theta activity in the frontal region provides time windows that allow neural information to be communicated across brain regions (Berger et al., 2019). Neural network distributions involving the frontal region were involved in WM processing, and the strength of prefrontal network activity decreased in patients with TLE during a WM task, which was associated with the task performance (Vlooswijk et al., 2011), in accordance with our results. Kaminski et al. (2019) evaluated EEG activity transmission during a WM task by measuring the full frequency DTF and short-time DTF and found that functional connectivity within the frontal region and between the frontal and posterior parietal regions increased, which also supports our results. During a WM task, the increased integration between cognitive networks and task-related non-cognitive network contributes to increased accuracy, indicating that increased communications among brain networks might underlie successful WM utilization (Cohen et al., 2014). Interestingly, information flows within and between brain networks through the synchronous activity of neural oscillations (Violante et al., 2017).

Cognitive impairment is a common comorbidity observed in patients with TLE, and ~80% of patients with TLE suffer from impairments in one or more cognitive domains, most frequently in language or memory (Winston et al., 2013; Allone et al., 2017; Rodríguez-Cruces et al., 2018; Chauvière, 2019; Reyes et al., 2019). Evidence suggests that WM is impaired in patients with TLE (Abrahams et al., 1999; Wagner et al., 2009; Black et al., 2010). In our results, patients with TLE performed the WM task with longer RT and lower ACC compared with the healthy controls. A study by Campo et al. compared magnetoencephalography (MEG) data between patients with TLE and healthy controls during the WM encoding phase and demonstrated reduced ACC among patients with TLE (Campo et al., 2009). Wagner et al. conducted a WM study including 96 patients with TLE and found that patients with unilateral TLE displayed WM deficits, irrespective of the affected hemisphere (Wagner et al., 2009).

Several factors may contribute to cognitive impairments in patients with TLE, including the underlying pathology, the impacts of seizure frequency and type, the age at seizure onset, disease duration, and treatment with ASMs (Helmstaedter et al., 2003; Elger et al., 2004; Fujikawa, 2005; Helmstaedter and Kockelmann, 2006; Hermann et al., 2006; Hermann and Seidenberg, 2007; Bell et al., 2011; Helmstaedter and Witt, 2017; Witt and Helmstaedter, 2017). Quantitative MRI studies have indicated widespread abnormalities across cortical and subcortical structures in patients with TLE, including the hippocampus, amygdala, fornix, parahippocampus, thalamus, basal ganglia, and neocortical regions (Keller and Roberts, 2008; Dabbs et al., 2012; Tai et al., 2018). Patients with TLE also exhibit microstructural abnormalities in the white matter tracts and decreased global network densities (Reyes et al., 2019). Cognitive impairments in patients with TLE may be associated with abnormalities in the underlying brain structures (Bell et al., 2011; Campo et al., 2013; Stretton et al., 2013; Winston et al., 2013). However, not all patients with TLE exhibit cognitive impairment, and patients with TLE who displayed generalized cognitive abnormalities showed widespread cortical and subcortical structural impairments, whereas patients with TLE who displayed near-normal cognitive functions showed minimal structural abnormalities (Hermann et al., 2007; Dabbs et al., 2009; Reyes et al., 2019). In our study, theta network strength was decreased in the TLE-WM group, compared with Con and TLE-N groups, despite a lack of significant differences in any examined clinical features between the TLE-WM group and the TLE-N group, whereas no significance in theta network activity was observed between the TLE-N group and the Con group, which was in agreement with previous studies (Hermann et al., 2007; Reyes et al., 2019). In regard to seizure frequency, a recent research of animal model showed that seizure frequency had a negative influence in working memory performance (Wolf et al., 2016). Seizure could affect the inhibitory-excitatory balance between brain regions, which could increase risk of cognitive impairment, and secondary damage due to hypoxia or brain trauma due to severe falls may result in irreversible cognitive impairment (Helmstaedter and Witt, 2017).

It was reported that WM performance in patients with TLE may be affected by anti-seizure medication (ASM; Smith et al., 2006). A study using a double-blind, randomized crossover design compared the cognitive effects of LTG and TPM in 47 healthy volunteers, and found that Lamotrigine produces significantly fewer untoward cognitive and behavioral effects compared to topiramate (TPM) at the dosages (Meador et al., 2005). Oxcarbazepine was reported without significant adverse impact on cognition, and Levetiracetam has a positive effect on working memory performance (Levisohn et al., 2009; Milovan et al., 2010; Operto et al., 2019). The ASM could reduce neuronal irritability while inhibiting neuronal excitability (Meador, 2002). Nevertheless, according to recent reviews and researches that cognitive impairments are present already before the ASM treatment (Elger et al., 2004; Hermann et al., 2007; Witt and Helmstaedter, 2017). Interestingly, TLE patients with cognitive impairment may also be at increased risk of cognitive impairments progression (Hermann et al., 2007).

In the current study, we evaluated theta network activity in patients with TLE who presented different WM task performance abilities and found that patients with impaired cognitive function displayed altered theta network activity compared with patients who displayed near-normal cognitive function, despite similarities in clinical features. Our results indicated that patients with TLE could not be reliably distinguished according to clinical features, and theta network analysis may represent a valid means for distinguishing differences in cognitive function and predicting cognitive outcomes among patients with TLE.

However, our study had several limitations. First, when exploring the aspect of education, no significant difference was observed between patients with TLE patients and healthy controls, whereas the education level of patients with TLE who displayed impaired task performance was lower than that of patients with TLE who displayed normal performance. Poor cognitive function may result in less education, which may, in turn, lead to worse cognitive function. Further studies remain necessary to clarify the relationships between TLE, education, and cognitive impairment. Second, patients with TLE in our study were not distinguished according to laterality, and differences in neural network mechanisms associated with WM deficit in patients with left vs. right TLE warrant a more detailed analysis. Third, further studies with regard to the gender differences in WM encoding in patients with TLE are still needed.

## Data Availability Statement

The original contributions presented in the study are included in the article/Supplementary Material, further inquiries can be directed to the corresponding author/s.

## Ethics Statement

The studies involving human participants were reviewed and approved by the Ethics Committee of the Tianjin Medical University General Hospital. The patients/participants provided their written informed consent to participate in this study.

## Author Contributions

YS and LP designed the study. LP, JB, YW, LZ, and XQ were involved in collection of data. LP, YW, DG, XZ, MD, PY, and GW were involved in analyses of the data with supervision of YS. LP drafted the paper under supervision of YS. All authors have read and approved the final manuscript, critically revised the manuscript, and have made substantial contributions to the design and concept of this study.

## Funding

This work was supported by the Tianjin Natural Science Foundation Beijing-Tianjin-Hebei Special Project (18JCZDJC44800) and Tianjin 131 Innovative Talents Team Training Project in 2016. This work was also supported by the Key Medical Discipline Construction Project of Tianjin, Science and Technology Project of Tianjin Medical Health Commission (TJWJ2021MS001), Tianjin Key Research and Development Plan, Key Project of Science and Technology Support (20YFZCSY00010).

## Conflict of Interest

The authors declare that the research was conducted in the absence of any commercial or financial relationships that could be construed as a potential conflict of interest.

## Publisher's Note

All claims expressed in this article are solely those of the authors and do not necessarily represent those of their affiliated organizations, or those of the publisher, the editors and the reviewers. Any product that may be evaluated in this article, or claim that may be made by its manufacturer, is not guaranteed or endorsed by the publisher.
