# Corrigendum: Alterations in Neural Networks During Working Memory Encoding Related to Cognitive Impairment in Temporal Lobe Epilepsy

**DOI:** 10.3389/fnhum.2022.883077

**Published:** 2022-03-18

**Authors:** Liping Pan, Yakun Wu, Jie Bao, Dandan Guo, Xin Zhang, Jiajing Wang, Meili Deng, Peiran Yu, Gaoxu Wei, Lulin Zhang, Xiao Qin, Yijun Song

**Affiliations:** ^1^General Medicine Department, Tianjin Medical University General Hospital, Tianjin, China; ^2^Department of Neurology, Tianjin Medical University General Hospital, Tianjin, China; ^3^Department of Neurology, Tangshan Gongren Hospital, Tangshan, China; ^4^Department of Rehabilitation Medicine, Tianjin Medical University General Hospital, Tianjin, China; ^5^School of Basic Medical Science, Tianjin Medical University, Tianjin, China; ^6^Department of Neurology, The First Affiliated Hospital of Fujian Medical University, Fuzhou, China; ^7^Key Laboratory of Central Nerve Injury Repair and Regeneration, Ministry of Education, Tianjin Neurological Institute, Tianjin, China

**Keywords:** working memory, encoding, temporal lobe epilepsy, theta, neural network

In the original article, we neglected to include the funders, “Key Medical Discipline Construction Project of Tianjin, Science and Technology Project of Tianjin Medical Health Commission (TJWJ2021MS001), Tianjin Key Research and Development Plan, Key Project of Science and Technology Support (20YFZCSY00010).”

The authors apologize for this error and state that this does not change the scientific conclusions of the article in any way. The original article has been updated.

## Publisher's Note

All claims expressed in this article are solely those of the authors and do not necessarily represent those of their affiliated organizations, or those of the publisher, the editors and the reviewers. Any product that may be evaluated in this article, or claim that may be made by its manufacturer, is not guaranteed or endorsed by the publisher.

