# Supplementary material for: Alterations in Neural Networks During Working Memory Encoding Related to Cognitive Impairment in Temporal Lobe Epilepsy
Source: Front Hum Neurosci. 2022 Jan 5;15:770678. doi: 10.3389/fnhum.2021.770678 (PMC8766724; doi:10.3389/fnhum.2021.770678)
Supplement: Supplementary file 1 [file Data_Sheet_1.PDF]

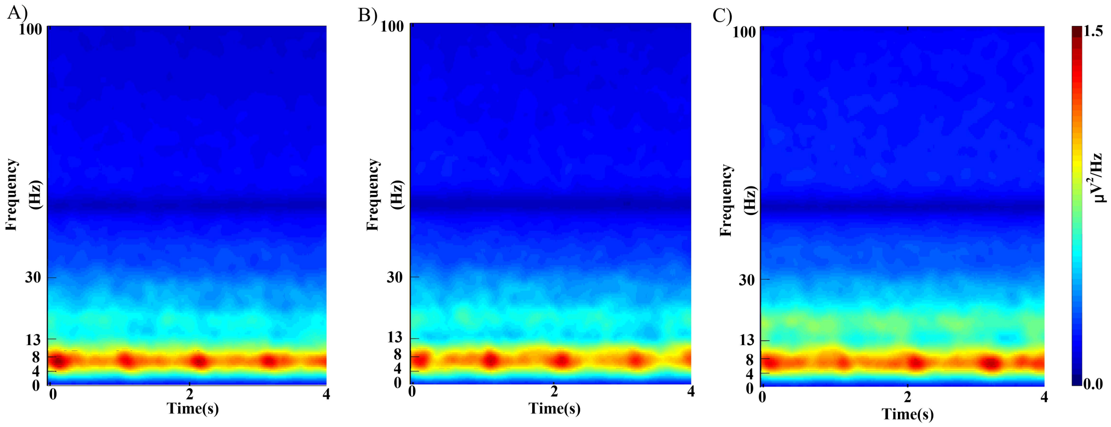

Suppl.Fig. Time-frequency representations of the Fz channel across frequency bands throughout WM encoding phase. A) Averaged time-frequency representations of power in the Con group. B) Averaged time-frequency representations of power in the TLE-N group. C) Averaged time-frequency representations of power in the TLE-WM group. Power is indicated by color. Strong theta activityy (4 – 8 Hz) was visible during the WM encoding phase.
